# Supplementary material for: Proteasome-Mediated Regulation of GATA2 Expression and Androgen Receptor Transcription in Benign Prostate Epithelial Cells
Source: Biomedicines. 2022 Feb 17;10(2):473. doi: 10.3390/biomedicines10020473 (PMC8962351; doi:10.3390/biomedicines10020473)
Supplement: Supplementary file 1 [file biomedicines-10-00473-s001.zip › biomedicines-1574326-supplementary.pdf]

Supplementary Materials

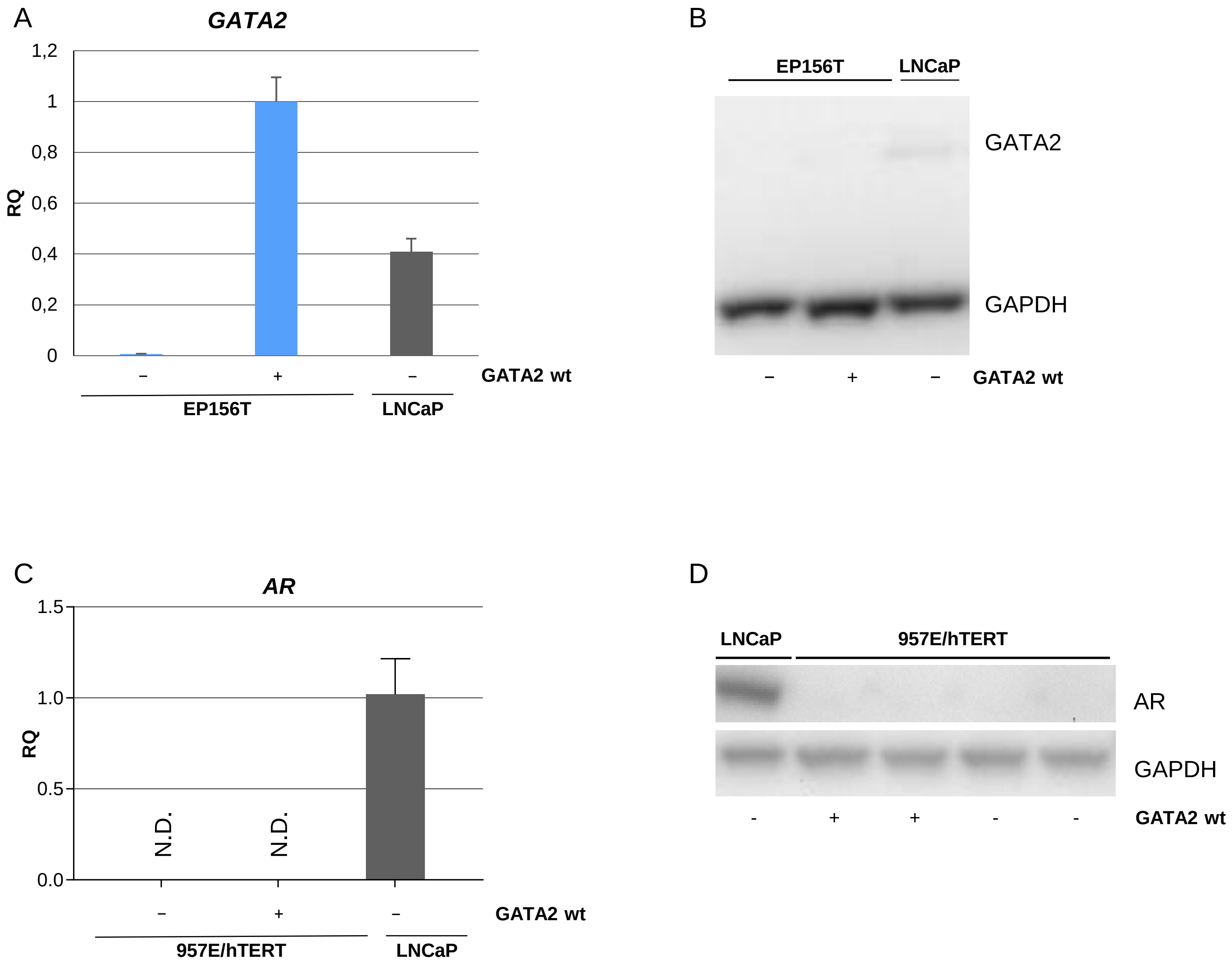

Figure S1. A) RT-qPCR and B) Western blot of exogenous GATA2 in EP156T cells. C) RT-qPCR and D) Western blot of AR in 957E/hTERT-GATA2 cells. Error bars represent standard error of the mean expression level (RQ) based on the RQmin/max of 95% confidence level. N.D. = not detected.

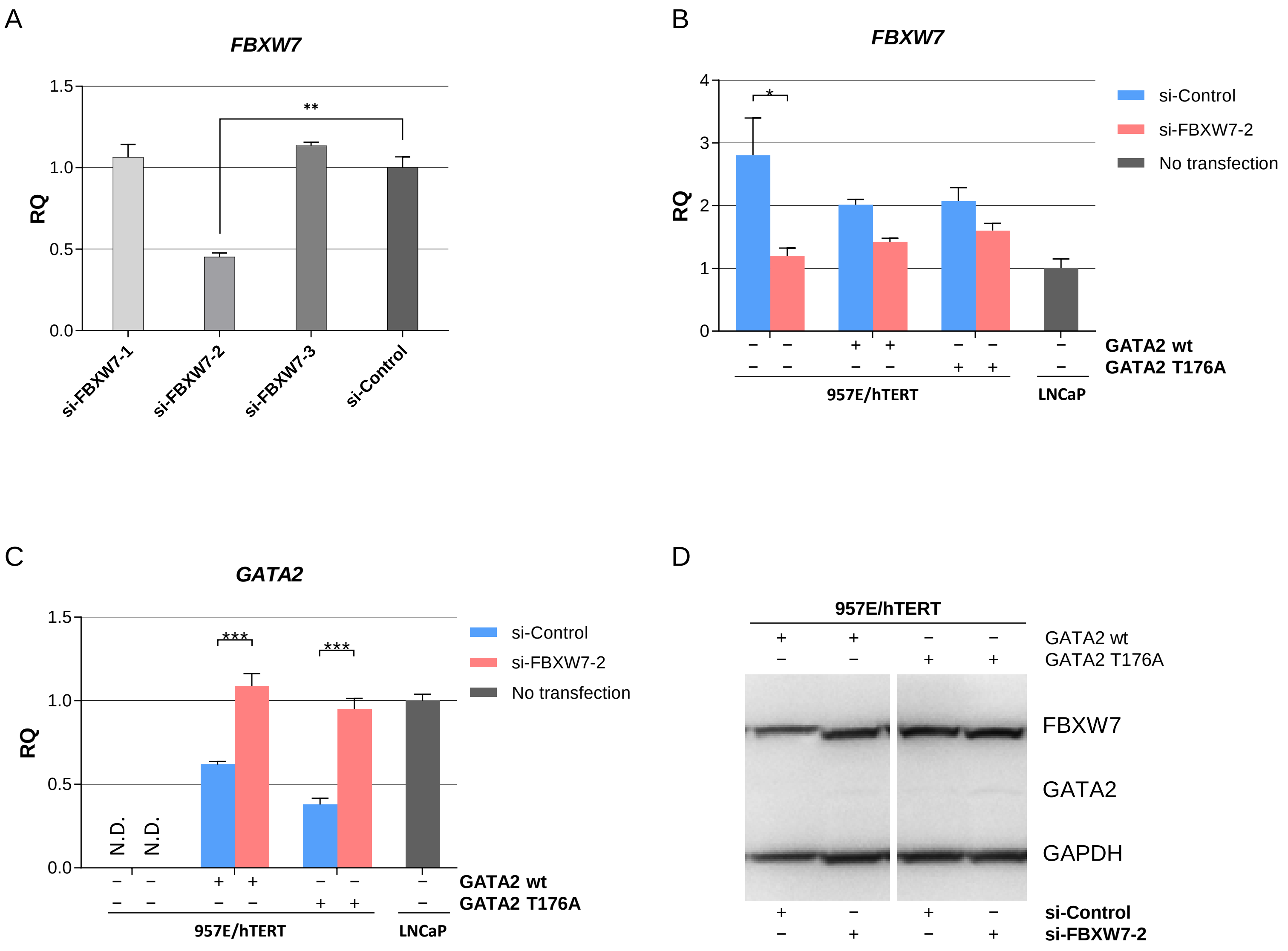

Figure S2. A) qPCR of 957E/hTERT cells transfected with siRNAs for FBXW7 or control siRNA (si-control) for 24 hours. B) and C) 957E/hTERT cells with stable transduction of GATA2 wt or GATA2 T176A and transfected with siRNA for FBXW7 (si-FBXW7-2) or control siRNA (si-control) for 48 hours. D) Western blot of 957E/hTERT cells with stable transduction of GATA2 wt or GATA2 T176A and transfected with siRNA for FBXW7 (si-FBXW7-2) or control siRNA (si-control) for 48 hours. Error bars represent standard error of the mean expression level (RQ) based on the RQmin/max of 95% confidence level. N.D.= not detected. *$p \leq 0.05$, **$p \leq 0.01$, ***$p \leq 0.001$ by using one-way ANOVA followed by Sidak's multiple comparisons test with 95% confidence interval.
